# Concurrent Stereotactic Radiotherapy and Immune Checkpoint Inhibitors for Multiple Brain Metastases in Non‐Small Cell Lung Cancer ‐ A Multi‐Center Retrospective Analysis

**DOI:** 10.1002/cns.71105

**Published:** 2026-08-25

**Authors:** Xiaoliang Wang, Yanan Zheng, Aimin Wang, Xiaolan Zhao, Kai Guan, Qiong Huang, Jiali Du

**Affiliations:** ^1^ Department of Radiotherapy The Third Hospital of Zhangzhou Zhangzhou Fujian China; ^2^ Department of Oncology The Third Hospital of Zhangzhou Zhangzhou Fujian China

**Keywords:** brain metastases, concurrent therapy, immune checkpoint inhibitors, non‐small cell lung cancer, stereotactic radiotherapy

## Abstract

**Background:**

This study aimed to evaluate the efficacy and safety of concurrent versus sequential stereotactic radiotherapy (SRT) and immune checkpoint inhibitors (ICIs) in non‐small cell lung cancer (NSCLC) patients with multiple brain metastases (BM).

**Methods:**

We retrospectively analyzed 198 NSCLC patients with BM who underwent SRT and ICIs treatment between 2017 and 2024. Of 136 eligible patients, were assigned to two cohorts and subcohorts through propensity score matching. The concurrent cohort consisted of 68 patients receiving ICIs within 14 days of SRT (post‐SRT 34 cases and pre‐SRT 34 cases). The sequential cohort included another 68 patients undergoing sequential treatment > 14 days apart (post‐SRT1 31 cases and pre‐SRT1 37 cases). Overall survival (OS) and intracranial progression‐free survival (iPFS) were the primary endpoints. Secondary endpoints were intracranial objective response rate (iORR), disease control rate (DCR) and adverse events (AEs).

**Results:**

Concurrent group achieved significantly superior median OS (24.5 months vs. 18.3 months, HR = 0.68, 95% CI 0.49–0.94, *p* = 0.001) and median iPFS (14.2 months vs. 9.8 months, HR = 0.62, 95% CI 0.45–0.86, *p* < 0.001) compared to the sequential group. Subgroup analysis, the post‐SRT group showed significantly improved median OS (27.1 months vs. 17.2, 19.2, 13.2 months, *p* < 0.05) and median iPFS (20.1 months vs. 10.5, 12.1, 5.3 months, *p* < 0.05) compared to the other three groups (pre‐SRT, post‐SRT1 and pre‐SRT1). Multivariate analysis revealed that a treatment interval ≤ 14 days was an independent protective factor for both iPFS (*p* = 0.004) and OS (*p* = 0.019). Subgroup analysis demonstrated that a treatment interval ≤ 7 days was superior to an interval of 8–14 days or > 14 days. Furthermore, ICIs following SRT was associated with improved OS (*p* = 0.003) and iPFS (*p* < 0.001). No significant differences were observed in radionecrosis (RN) and AEs.

**Conclusion:**

Concurrent SRT with ICI, especially ICIs following SRT, provides superior survival outcomes for NSCLC patients with multiple BM, without increasing AEs. The timing of therapy is a critical factor influencing the efficacy of the combined regimen.

## Graphical abstract

Initiating ICIs within 14 days post SRT, especially at a short interval after radiotherapy, markedly extends OS and iPFS, with no elevated risks of RN or adverse events.

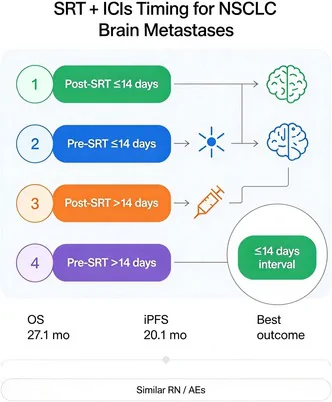

**Abbreviations**
Ad: adenocarcinoma
AEs: adverse events
ALK: anaplastic lymphoma kinase
BBB: blood–brain barrier
BM: brain metastases
CNS: central nervous system
CR: complete response
DAMPs: damage‐associated molecular patterns
DCR: disease control rate
ECM: extracranial metastases
EGFR: epidermal growth factor receptor
GPA: graded prognostic assessment
GTV: gross tumor volume
HFSRT: high‐fraction stereotactic radiotherapy
ICI: immune checkpoint inhibitor
iORR: intracranial objective response rate
iPFS: intracranial progression‐free survival
irAEs: immune‐related adverse events
KPS: Karnofsky Performance Status
LMD: leptomeningeal disease
No: number
NSCLC: non‐small cell lung cancer
OS: overall survival
PD‐L1: programmed death‐ligand 1
PGTV: planning gross tumor volume
post‐SRT: post stereotactic radiation therapy
PR: partial response
pre‐SRT: pre‐stereotactic radiation therapy
PSM: propensity score‐matching
RANO‐BM: Neuro‐Oncology Brain Metastases
RN: radionecrosis
SCC: squamous cell carcinoma
SD: stable disease
SRT: stereotactic radiotherapy
TME: tumor microenvironment
TRP: Treatment‐related pneumonitis
WBRT: whole‐brain radiotherapy

## 1 Introduction

Lung cancer represents the leading cause of cancer‐related mortality globally, with NSCLC accounting for approximately 85% of all lung cancer cases [1]. BM is among the most common sites of distant metastasis in NSCLC. Approximately 25%–30% of patients with advanced NSCLC present with BM at initial diagnosis, and this proportion can rise to 50% as the disease progresses [2]. The development of BM not only severely impacts patients' neurological function and quality of life but is also a major factor leading to poor prognosis.

Historically, treatment modalities for BM have included surgery, whole‐brain radiotherapy (WBRT), and SRT. As a precise radiation technique, SRT delivers high doses of radiation to tumor lesions while minimizing damage to surrounding normal brain tissue and has become the standard of care for patients with a limited number (1–4) of BM [3]. However, SRT alone has limited efficacy in controlling intracranial disease and preventing new lesions, particularly in patients with multiple BM.

With the development of cancer immunotherapy, ICIs such as PD‐1/PD‐L1 inhibitors, have demonstrated significant efficacy in the treatment of NSCLC. Studies indicated that ICIs also exhibit activity against NSCLC BM, although the objective response rate for monotherapy is typically only 20%–40% [4]. In recent years, the combination of radiotherapy and immunotherapy has emerged as a major research focus. The theoretical rationale bases on the ability of radiotherapy to promote tumor antigen release and presentation, modify the tumor microenvironment (TME), and thereby enhance the response to immunotherapy; concurrently, immunotherapy may amplify the abscopal effect of radiotherapy [5].

Despite these theoretical advantages, the optimal timing of SRT in combination with ICIs remains a critical unresolved issue in clinical practice. Some studies suggest that the treatment interval may influence both efficacy and toxicity. Preclinical research indicates that initiating immunotherapy shortly after radiotherapy (typically within one week) may maximize the synergistic anti‐tumor effects [6]. The median half‐life of ipilimumab is about 14 days, and nearly 20 days for trelilizumab, sintilimab, tislelizumab, nivolumab and pembrolizumab, versus atezolizumab is 27 days. Moreover, several earlier investigations have demonstrated that delivering ICIs shortly after SRT leads to superior synergistic efficacy [7, 8, 9, 10]. Given the above considerations, we set a two‐week cutoff point in this research. Despite obtaining statistically meaningful results, the influence of radiation therapy on the temporal patterns of T‐cell activation and tumor infiltration has not been fully elucidated. Furthermore, the ideal clinical order for delivering SRS/SRT alongside immune checkpoint blockade therapy has yet to reach a widely agreed standard.

Consequently, this multicenter retrospective study was designed to compare the clinical efficacy and safety between concurrent and sequential regimens of SRT plus ICIs for NSCLC patients complicated with multiple brain metastases. We performed four pairwise subgroup analyses, stratifying patients into those receiving ICIs within 14 days before radiotherapy versus those receiving ICIs beyond 14 days pre‐SRT, and those administered ICIs within 14 days post‐radiotherapy versus those given ICIs more than 14 days after SRT. The findings of this study may serve as an evidence‐based reference for refining clinical treatment strategies.

## 2 Materials and Methods

### 2.1 Study Design and Patient Selection

**FIGURE 1:**
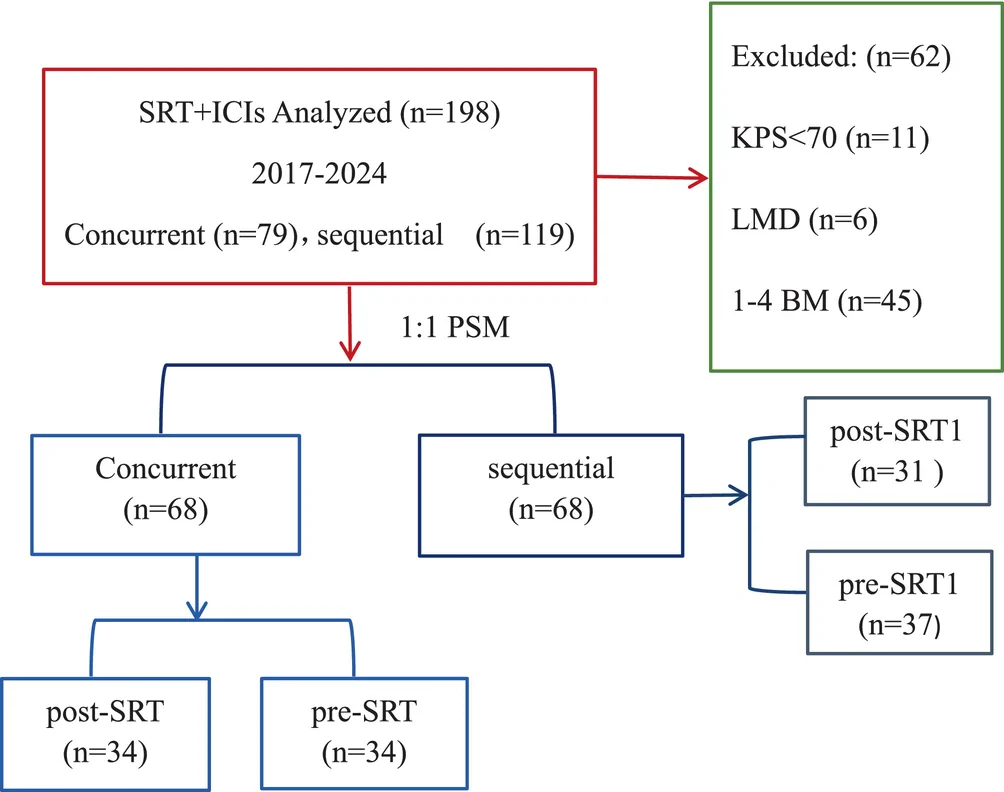
198 patients receiving SRT plus ICIs from 2017 to 2024 were enrolled at baseline (concurrent group: *n* = 79; sequential group: *n* = 119). A total of 62 cases were excluded owing to KPS < 70 (*n* = 11), leptomeningeal metastases (*n* = 6), and 1–4 brain metastases (*n* = 45). After 1:1 propensity score matching, 68 patients remained in each arm. The concurrent group was split into post‐SRT (*n* = 34) and pre‐SRT (*n* = 34) subgroups; the sequential group was divided into post‐SRT1 (*n* = 31) and pre‐SRT1 (*n* = 37) subgroups.

As a multicenter retrospective investigation, this study retrieved data from the medical record systems of three collaborating hospitals, with ethical approval acquired from the institutional review board of each respective institution. We retrospectively analyzed clinical data of patients diagnosed with non‐small cell lung cancer (NSCLC) complicated by brain metastases. A total of 198 eligible patients were enrolled in this cohort study, including 16 cases from the Third Hospital of Zhangzhou, 154 from Xiamen Changgung Hospital, and 28 from the 73rd Group Army Hospital. All participants were consecutively recruited from January 2017 to December 2024. Key inclusion criteria were: (1) Histologically confirmed as NSCLC, (2) Radiologically confirmed diagnosis of BM, (3) Treatment with SRT + ICIs for BM, (4) Availability of baseline and follow‐up MRI brain scans and clinical records. Key exclusion criteria were: (1) Prior whole‐brain radiotherapy (WBRT), (2) Incomplete treatment records or inadequate imaging follow‐up (< 3 months), (3) Received < 2 cycles ICIs, (4) Patients who underwent SRT before or during targeted therapy were excluded. The raw dataset of the 198 initially enrolled patients exhibited bias before propensity score matching (PSM). Therefore, patients with KPS < 70 (*n* = 11), leptomeningeal metastasis (*n* = 6), and 1–4 brain metastases (*n* = 45) were excluded to remove confounding variables interfering with baseline equilibrium. A 1:1 propensity score matching was performed between the two groups to reduce dataset bias. 136 eligible patients were divided into two groups based on the treatment interval between SRT and ICIs: the concurrent group (*n* = 68, SRT + ICIs interval ≤ 14 days; subgroups: post‐SRT 34 cases and pre‐SRT 34 cases) and the sequential group (*n* = 68, SRT + ICIs interval > 14 days; subgroups: post‐SRT1 31 cases and pre‐SRT1 37 cases). see (Figure 1).

### 2.2 Treatment Protocols

SRT: Treatment planning utilized thin‐slice (≤ 1 mm) MRI fused with CT simulation scans. Dose selection adhered to established guidelines (RTOG 90–05, QUANTEC), considering lesion volume, location, and prior treatments. Target volume delineation (GTV = visible metastasis; PGTV = GTV + 2 mm margin) followed institutional protocol. The prescribed dose is 30–36 Gy/5–6 fractions (high‐fraction stereotactic radiotherapy, HFSRT).

ICIs: Patients received standard FDA‐approved doses and schedules (pembrolizumab 200 mg IV q3w, nivolumab 240 mg IV q2w or 480 mg IV q4w, atezolizumab 1200 mg IV q3w, ipilimumab 3 mg/kg IV q3w for up to 4 doses, often combined with nivolumab). Concurrent Group: Initiation of the first ICI dose occurred within 14 days before or after the start of SRT. Sequential Group: The start of ICIs therapy occurred more than 14 days before or after the start of SRT.

### 2.3 Follow‐Up

Patients were generally assessed clinically and with MRI brain every 2–3 months post‐treatment. Response was assessed using the Response Assessment in Neuro‐Oncology Brain Metastases (RANO‐BM) criteria [7]. The primary outcomes included iPFS and OS. OS: Time from date of SRT start to death from any cause or last follow‐up. Intracranial Progression‐Free Survival (iPFS): Time from SRT start to intracranial progression (new BM, progression of non‐target existing BM, or progression of a treated lesion per RANO‐BM) or death. Secondary outcome measures included intracranial objective response rate (iORR), disease control rate (DCR), and treatment‐related toxicity. iORR was defined as the proportion of patients with complete response (CR) and partial response (PR), while DCR was defined as the proportion of patients with CR, PR, and stable disease (SD). The safety assessment included acute and late toxic reactions, graded in accordance with the CTCAE 5.0 criteria. Particular attention was given to the occurrence of radiation‐induced brain necrosis, immune‐related adverse events (irAEs), and treatment‐related pneumonia.

### 2.4 Statistical Analysis

**FIGURE 2:**
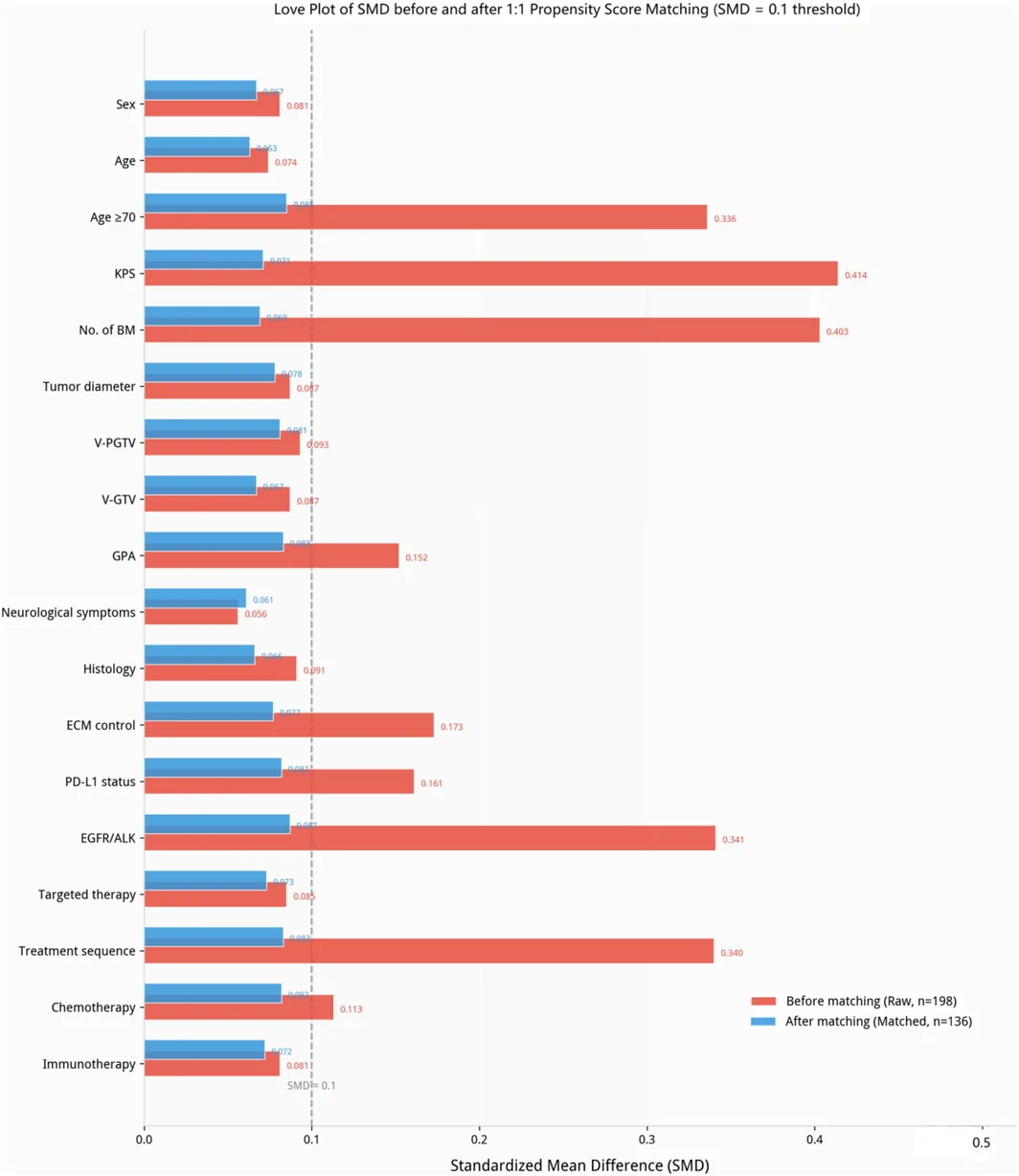
Love plot illustrating standardized mean differences (SMDs) of all baseline covariates before and after 1:1 propensity score matching (PSM). Red horizontal bars indicate SMD values in the original unmatched cohort consisting of 198 patients, while blue horizontal bars represent SMD values in the 1:1 PSM‐matched cohort with a sample size of 136 patients. The vertical dashed gray line marks the conventional balance threshold of SMD = 0.1.

Statistical analyses were performed using SPSS software (version 25.0; IBM Corp, Armonk, NY, USA). For categorical covariates, between‐group differences were evaluated via standardized mean differences (SMDs) computed in SPSS. Survival analysis was conducted using the Kaplan–Meier to estimate survival rates, with between‐group comparisons assessed by the log‐rank test. Multivariable Cox proportional hazards regression modeling was employed to identify independent prognostic factors. Patients were divided into two cohorts through 1:1 propensity score matching (PSM): SRT combined with immune checkpoint inhibitors (ICIs) (SRT + ICIs) the concurrent group and sequential group. Propensity score matching balanced potential prognostic factors via multivariate logistic regression incorporating: age (< 70 vs. ≥ 70 years), number of BM (1–4, 5–10 vs. 11–22), GPA score (0–1.0/1.5–2.0/2.5–3.0), extracranial disease control status at SRT initiation (controlled/uncontrolled), PD‐L1 expression (50–100%/1–49%/negative or unknown), EGFR/ALK status (positive/negative), and Therapeutic sequence (ICIs after SRT vs. ICIs before SRT). Matching used a caliper width of 0.2 times the logit standard deviation of the propensity score without replacement (concurrent group, *n* = 68; sequential group, *n* = 68). See (Table 1) and (Figure 2). A two‐sided *p*‐value < 0.05 was considered statistically significant.

**TABLE 1:** Patient baseline characteristics in the raw and matched datasets.

Characteristics	Raw dataset (*n* = 198)			Matched dataset (*n* = 136)		
	Concurrent (%)	Sequential (%)	SMDs	Concurrent (%)	Sequential (%)	SMDs
Sample size, No	79	119		68	68	
Sex						0.067
Male	47 (59.5)	69 (58.0)	0.081	42 (61.8)	44 (64.7)	
Female	32 (40.5)	50 (42.0)		26 (38.2)	24 (35.3)	
Age, year	62 (29–87)	64 (31–86)	0.074	63 (30–84)	62 (33–85)	0.063
< 70	48 (60.8)	54 (45.4)	0.336	44 (64.7)	43 (63.2)	0.085
≥ 70	31 (39.2)	84 (54.6)		24 (35.3)	25 (36.8)	
KPS			0.414			0.071
≥ 80	56 (70.9)	77 (64.7)		49 (72.1)	47 (69.1)	
< 80	23 (29.1)	42 (35.3)		19 (27.9)	21 (30.9)	
No. of BM at BM diagnosis			0.403			0.069
1–4	8 (10.1)	37 (31.1)				
5–10	44 (55.7)	49 (41.2)		43 (63.2)	42 (61.8)	
11–22	27 (34.2)	33 (27.7)		25 (36.8)	26 (38.2)	
Diameter of the largest tumor, mm						0.078
≤ 20	49 (62.0)	69 (58.0)	0.087	44 (64.7)	43 (63.2)	
> 20	30 (38.0)	50 (42.0)		24 (35.3)	25 (36.8)	
V‐PGTV (cm^3^)	30.78 (10.77–109.33)	31.83 (11.24–116.42)	0.093	29.24 (12.78–85.21)	29.83 (13.32–86.41)	0.081
V‐GTV (cm^3^)	12.33 (5.35–35.68)	13.53 (5.52–39.48)	0.087	11.58 (5.35–29.21)	11.76 (5.52–28.98)	0.067
GPA						
0–1.0	12 (15.2)	30 (25.2)	0.152	11 (16.2)	13 (19.1)	0.083
1.5–2.0	30 (38.0)	54 (45.4)		26 (38.2)	25 (36.8)	
2.5–3.0	37 (46.8)	35 (29.4)		31 (45.6)	30 (44.1)	
3.5–4.0	0	0		0	0	
Neurological symptoms positive	26 (32.9)	45 (37.8)	0.056	21 (30.8)	23 (33.8)	0.061
Histology (%)						0.066
SCC	29 (36.7)	54 (45.4)	0.091	26 (38.8)	28 (41.2)	
Ad	50 (63.3)	65 (54.6)		42 (61.2)	40 (58.8)	
ECM control at SRT initiation						0.077
Controlled	48 (60.8)	53 (44.5)	0.173	40 (58.8)	42 (61.8)	
Uncontrolled	31 (39.2)	66 (55.5)		28 (41.2)	26 (38.2)	
PD‐L1 status						0.082
50%–100%	11 (13.9)	13 (10.9)	0.161	11 (16.2)	12 (17.6)	
1%–49%	29 (36.7)	25 (21.0)		28 (41.2)	24 (35.3)	
Negative	39 (49.4)	81 (68.1)		29 (42.6)	32 (47.1)	
Mutations						0.087
EGFR/ALK	36 (45.6)	30 (25.2)	0.341	31 (45.6)	29 (42.6)	
Negative/other	43 (54.4)	89 (74.8)		37 (54.4)	39 (57.4)	
Targeted therapy before ICIs						
Osimertinib	15 (41.7)	10 (33.3)	0.085	12 (38.7)	11 (37.9)	0.073
Almonertinib	3 (8.3)	4 (13.3)		3 (9.7)	4 (13.8)	
Gefitinib	7 (19.4)	8 (26.7)		7 (22.6)	6 (20.7)	
Erlotinib	3 (8.3)	2 (6.7)		3 (9.7)	4 (13.8)	
Afatinib	4 (11.1)	3 (10.0)		4 (12.9)	2 (6.9)	
Lorlatinib	2 (5.6)	2 (6.7)		1 (3.2)	2 (6.9)	
Brigatinib	1 (2.8)	1 (3.3)		1 (3.2)	0	
Alectinib	1 (2.8)	0				
Treatment sequence (ICIs)						
ICIs post‐SRT	42 (53.2)	38 (31.9)	0.340	34 (50.0)	31 (45.6)	0.083
ICIs pre‐SRT	37 (46.8)	81 (68.1)		34 (50.0)	37 (54.4)	
Chemotherapy after ICIs and Target						
Yes	65 (82.3)	80 (67.2)	0.113	55 (80.9)	57 (83.8)	0.082
No	14 (17.7)	39 (32.8)		13 (19.1)	11 (16.2)	
Immunotherapy						
Pembrolizumab	28 (35.4)	34 (28.6)	0.081	25 (36.8)	26 (38.2)	0.072
Atezolizumab	15 (19.0)	31 (26.1)		14 (20.6)	15 (22.0)	
Nivolumab	13 (16.5)	6 (5.0)		11 (16.2)	10 (14.7)	
Trelilizumab	8 (10.1)	25 (21.0)		6 (8.8)	5 (7.4)	
Sintilimab	9 (11.4)	19 (15.9)		8 (11.7)	7 (10.3)	
Tislelizumab	6 (7.6)	4 (3.4)		4 (5.9)	5 (7.4)	

Abbreviations: Ad, adenocarcinoma; ALK, anaplastic lymphoma kinase; BM brain metastases, ECM, extracranial metastases; EGFR, epidermal growth factor receptor; GPA, graded prognostic assessment; ICI, immune checkpoint inhibitor; KPS, karnofsky performance status; No., number; PD‐L1, programmed death‐ligand 1; SCC, squamous cell carcinoma; SMDs, standardized mean differences SRT stereotactic radiotherapy; V‐PGTV, volume of vlanning gross tumor volume.

## 3 Results

### 3.1 Patient Clinical and Treatment Characteristics

We retrospectively analyzed 198 NSCLC patients with brain metastases (BM) treated with stereotactic radiotherapy (SRT) and immune checkpoint inhibitors (ICIs) between 2017 and 2024. After 1:1 PSM, 136 eligible patients with 952 BM lesions were included in the final analysis. These patients were assigned into concurrent group (*n* = 68, 464 BM; subgroup: post‐SRT 34 cases and pre‐SRT 34 cases) and sequential group (*n* = 68, 488 BM; subgroup: post‐SRT1 31 cases and pre‐SRT1 37 cases) (Figure 1). Baseline characteristics were well‐balanced between groups regarding age, sex, histological type, KPS, number of BM, maximum BM diameter, V‐GTV, V‐PGTV, GPA, present of neurological symptoms, PD‐L1 status, EGFR/ALK Mutations, Targeted therapy before ICIs, therapeutic sequence (ICIs), Chemotherapy after ICIs and Target, immunotherapy, and ECM control at SRT initiation (Table 1). However, some biased feature were found in the raw dataset, such as younger patients (SMDs = 0.336), a lower KPS (SMDs = 0.414), Oligometastasis (SMDs = 0.403), a worse GPA score (SMDs = 0.152), ECM control at SRT initiation (SMDs = 0.173), PD‐L1 status (SMDs = 0.161), Mutations (SMDs = 0.341), therapeutic sequence (ICIs) (SMDs = 0.340), and Chemotherapy after ICIs and Target (SMDs = 0.113) 0.136 patients were divided into two groups through 1:1 propensity score matched. Each character in the matched dataset was well‐balanced, as shown in (Table 1) and (Figure 2).

### 3.2 Survival Outcomes

**FIGURE 3:**
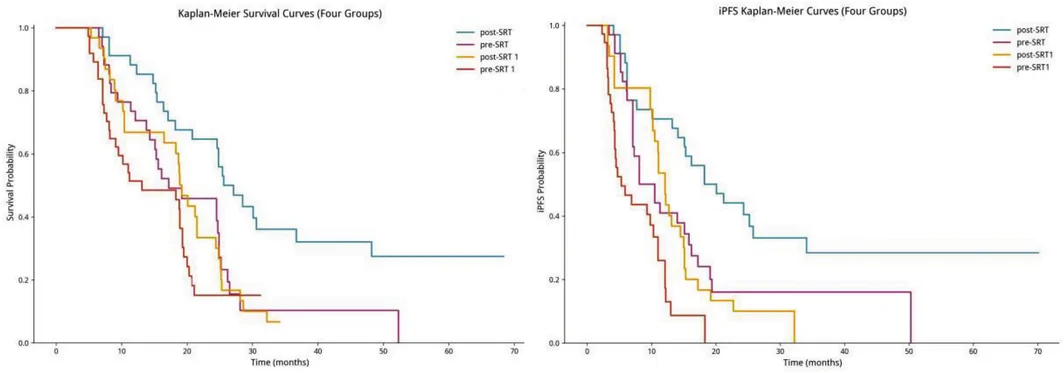
Kaplan–Meier survival curves for OS and iPFS comparing the concurrent SRT + ICIs group (post‐SRT versus. pre‐SRT) to the sequential SRT + ICIs group (post‐SRT versus. pre‐SRT) in the propensity score‐matched dataset. OS prognostic outcomes: Post‐SRT > post‐SRT1 ≈ pre‐SRT > pre‐SRT1; iPFS prognostic outcomes: Post‐SRT > post‐SRT1 ≈ pre‐SRT > pre‐SRT1.

With a median follow‐up of 26.3 months (range: 8.5–45.2 months), this study demonstrated that the concurrent group achieved significantly superior median OS (24.5 months vs. 18.3 months, HR = 0.68, 95% CI 0.49–0.94, *p* = 0.001) and median iPFS (14.2 months vs. 9.8 months, HR = 0.62, 95% CI 0.45–0.86, *p* < 0.001) compared to the sequential group. Subgroup analysis, the post‐SRT group of concurrent treatment demonstrated superior survival outcomes and iPFS. Median overall survival (OS) was markedly longer in the post‐SRT group than in the other three cohorts (pre‐SRT, post‐SRT1, pre‐SRT1), reaching 27.1 months versus 17.2 months, 19.2 months and 13.2 months, respectively (*p* = 0.006, 0.003, and < 0.001). No difference was observed between the pre‐SRT and post‐SRT1 groups (HR = 0.942, 95% CI 0.554–1.602, *p* = 0.824). The pre‐SRT1 group had the worst prognosis; however, comparisons with the pre‐SRT (*p* = 0.111) and post‐SRT1 (*p* = 0.176) groups failed to reach statistical significance, possibly due to limited sample size. The prognostic hierarchy for OS was: post‐SRT > post‐SRT1 ≈ pre‐SRT > pre‐SRT1. See the (Figure 3) and (Table 2).

**TABLE 2:** Pairwise Cox regression analyses across four groups (OS).

Concurrent (post‐SRT vs. pre‐SRT) versus sequential (post‐SRT1 vs. pre‐SRT1)	HR	95% CI	*p*
Post‐SRT vs. pre‐SRT	0.446	(0.252–0.788)	0.006
Post‐SRT vs. post‐SRT1	0.419	(0.235–0.745)	0.003
Post‐SRT vs. pre‐SRT1	0.334	(0.184–0.604)	< 0.001
Pre‐SRT vs. post‐SRT1	0.942	(0.554–1.602)	0.824
Pre‐SRT vs. pre‐SRT1	0.650	(0.383–1.104)	0.111
Post‐SRT1 vs. pre‐SRT1	0.693	(0.408–1.179)	0.176

Abbreviations: OS, overall survival; post‐SRT, post stereotactic radiation therapy; pre‐SRT, pre‐stereotactic radiation therapy; SRT, stereotactic radiotherapy.

Similarly, median iPFS was significantly improved in the post‐SRT group (20.1 months) relative to the pre‐SRT, post‐SRT1 and pre‐SRT1 groups (10.5 months, 12.1 months and 5.3 months, respectively; *p* = 0.013, 0.002, and *p* < 0.001) and a 52%–79.5% reduction in intracranial progression risk. Statistically significant differences were observed in both the pre‐SRT vs. pre‐SRT1 and post‐SRT1 vs. pre‐SRT1 pairwise groups (*p* = 0.006, 0.003), but there were no significant differences between the pre‐SRT and the post‐SRT1 groups. The pre‐SRT1 group exhibited the worst iPFS and differed significantly from all other cohorts, with a median iPFS of only 5.3 months. The prognostic hierarchy for iPFS was: post‐SRT > post‐SRT1 ≈ pre‐SRT > pre‐SRT1. Although this trend was consistent with OS, intergroup disparities were more pronounced for iPFS, especially for the pre‐SRT1 group (Figure 3, Table 3).

**TABLE 3:** Pairwise Cox regression analyses across four groups (iPFS).

Concurrent (post‐SRT vs. pre‐SRT) versus sequential (post‐SRT1 vs. pre‐SRT1)	HR	95% CI	*p*
Post‐SRT vs. pre‐SRT	0.483	(0.272–0.855)	0.013
Post‐SRT vs. post‐SRT1	0.406	(0.228–0.723)	0.002
Post‐SRT vs. pre‐SRT1	0.205	(0.105–0.400)	< 0.001
Pre‐SRT vs. post‐SRT1	0.919	(0.539–1.565)	0.755
Pre‐SRT vs. pre‐SRT1	0.459	(0.264–0.799)	0.006
Post‐SRT1 vs. pre‐SRT1	0.423	(0.241–0.743)	0.003

Abbreviations: iPFS, intracranial progression‐free survival; post‐SRT, post stereotactic radiation therapy; pre‐SRT, pre‐stereotactic radiation therapy; SRT, stereotactic radiotherapy.

Multivariate analysis identified a treatment interval (time between SRT and ICI initiation) ≤ 14 days as an independent protective factor for both iPFS (HR 0.501, 0.443–0.581, *p* = 0.004) and OS (HR 0.653, 0.543–0.847, *p* = 0.019). Subgroup analysis further indicated that a treatment interval ≤ 7 days conferred superior OS (HR 0.796, 95% CI 0.773–0.951, *p* = 0.003) and iPFS (HR 0.732, 95% CI 0.573–0.905, *p* = 0.024) compared to intervals of 8–14 days. Administration of ICIs following SRT (concurrent group protocol) was associated with improved OS and iPFS compared to the pre‐SRT group (HR 0.445, 95% CI 0.252–0.788, *p* = 0.005; and HR 0.483, 95% CI 0.272–0.855, *p* = 0.012). Independent factors associated with significantly shortened survival included age ≥ 70 years (*p* = 0.032), KPS score < 80 (*p* = 0.013), No. of BM at BM diagnosis 11–22 (*p* = 0.038), lower GPA 0–1.0 (*p* = 0.008, 0.028), positive neurological symptoms (*p* = 0.017), SCC (*p* = 0.012), ECM uncontrolled at time of SRT (*p* = 0.041), PD‐L1 negative (*p* = 0.013), and no EGFR/ALK mutations (*p* = 0.007). Several variables associated with statistically significant improvements in iPFS were as follows: KPS ≥ 80 (*p* = 0.019), BM 5–10 (*p* = 0.033), Dmax ≤ 20 (*p* = 0.035), higher GPA 2.5–3.0 (*p* = 0.018), negative neurological symptoms (*p* = 0.030), positive PD‐L1 (*p* = 0.011), and EGFR/ALK mutations (*p* = 0.022) (see the Table 4 and Table 5).

**TABLE 4:** Overall survival.

All patients of 1:1 PSM *n* = 136						
Variable	Univariate analysis			Multivariate analysis		
	HR	95% CI	*p*	HR	95% CI	*p*
Age (< 70 vs. ≥ 70)	0.705	0.348–0.891	0.023	0.733	0.432–0.921	0.032
Sex (Male vs. Female)	1.014	0.752–1.681	0.741			
KPS at BM diagnosis						
≥ 80 vs.< 80	0.592	0.456–0.796	0.007	0.628	0.317–0.893	0.013
No. of BM at BM diagnosis						
5–10 vs. 11–22	0.882	0.711–0.978	0.042	0.878	0.741–0.972	0.038
Diameter of the largest tumor, mm						
≤ 20 vs.> 20	1.071	0.719–1.796	0.422			
GPA						
1.5–2.0 vs. 0–1.0	0.591	0.489–0.763	0.023	0.613	0.446–0.886	0.028
2.5–3.0 vs. 0–1.0	0.446	0.311–0.824	0.015	0.471	0.378–0.714	0.008
Neurological symptoms						
Negative vs. Positive	0.614	0.513–0.898	0.014	0.641	0.513–0.883	0.017
Histology (%)						
SCC vs. Ad	1.002	0.521–1.534	0.421	2.015	1.352–5.632	0.012
ECM Control at time of SRT						
Controlled vs. uncontrolled	0.732	0.585–0.981	0.046	0.583	0.453–0.705	0.041
PD‐L1 status						
50%–100% vs. Negative	0.445	0.323–0.811	< 0.001	0.573	0.221–0.886	0.013
1%–49% vs. Negative	0.803	0.657–1.512	0.765	1.091	0.679–1.544	0.238
Mutations						
EGFR/ALK vs. Negative/other	0.687	0.459–0.889	0.013	0.565	0.475–0.913	0.007
Treatment sequence (ICIs)						
Post‐SRT vs. pre‐SRT	0.733	0.551–0.913	0.021	0.501	0.340–0.739	0.003
Concurrent vs. Sequential	0.668	0.547–0.868	0.024	0.653	0.543–0.847	0.019
Concurrent						
Post‐SRT vs. pre‐SRT	0.721	0.465–0.813	0.023	0.445	0.252–0.788	0.005
Sequential						
Post‐SRT1 vs. pre‐SRT1	0.877	0.495–0.968	0.032	0.693	0.408–1.176	0.176
Concurrent SRT + ICIs						
≤ 7 days vs. 8–14 days	0.812	0.732–0.977	0.045	0.796	0.773–0.951	0.033

Abbreviations: Ad, adenocarcinoma; ALK, anaplastic lymphoma kinase; BM, brain metastases; ECM, extracranial metastases; EGFR, epidermal growth factor receptor; GPA, graded prognostic assessment; ICI, immune checkpoint inhibitor; KPS, karnofsky performance status; No., number; PD‐L1, programmed death‐ligand 1; SC, squamous cell carcinoma; SRT, stereotactic radiotherapy.

**TABLE 5:** Intracranial progression‐free survival.

All patients of 1:1 PSM *n* = 136						
Variable	Univariate analysis			Multivariate analysis		
	HR	95% CI	*p*	HR	95%	*p*
Age (< 70 vs. ≥ 70)	0.673	0.452–1.114	0.376			
Sex (Male vs. Female)	1.041	0.712–1.495	0.693			
KPS at BM diagnosis						
≥ 80 vs.< 80	0.789	0.597–0.971	0.028	0.752	0.612–0.912	0.019
No. of BM at BM diagnosis						
5–10 vs. 11–22	0.715	0.581–0.932	0.039	0.708	0.615–0.896	0.033
Diameter of the largest tumor, mm						
≤ 20 vs.> 20	0.763	0.611–0.941	0.041	0.715	0.631–0.893	0.035
GPA						
1.5–2.0 vs. 0–1.0	0.711	0.466–0.816	0.032	0.652	0.581–0.891	0.029
2.5–3.0 vs. 0–1.0	0.643	0.521–0.831	0.027	0.598	0.521–0.681	0.018
Neurological symptoms						
Negative vs. Positive	0.821	0.613–0.974	0.032	0.763	0.572–0.945	0.030
Histology (%)						
SCC vs. Ad	0.813	0.728–2.531	0.141			
ECM control at time of SRT						
Controlled vs. uncontrolled	0.787	0.611–1.029	0.125			
PD‐L1 status						
50%–100% vs. Negative	0.733	0.553–0.941	0.025	0.714	0.563–0.913	0.019
1%–49% vs. Negative	0.821	0.543–0.972	0.031	0.734	0.591–0.885	0.011
Mutations						
EGFR/ALK vs. Negative/other	0.511	0.345–0.916	0.012	0.458	0.276–0.896	0.022
Therapeutic sequence (ICIs)						
Post‐SRT vs. pre‐SRT	0.631	0.432–0.932	0.033	0.501	0.340–0.739	< 0.001
Concurrent vs. Sequential	0.713	0.528–0.915	0.015	0.501	0.443–0.581	0.004
Concurrent						
Post‐SRT vs. pre‐SRT	0.852	0.412–0.976	0.041	0.483	0.272–0.855	0.012
Sequential						
Post‐SRT1 vs. pre‐SRT1	0.883	0.389–0.981	0.046	0.423	0.241–0.743	0.002
Concurrent SRT + ICIs						
≤ 7 days vs. 8–14 days	0.817	0.513–0.923	0.036	0.732	0.573–0.905	0.024

Abbreviations: Ad, adenocarcinoma; ALK, anaplastic lymphoma kinase; BM, brain metastases; ECM, extracranial metastases; EGFR, epidermal growth factor receptor; GPA, graded prognostic assessment; ICI, immune checkpoint inhibitor; KPS, Karnofsky performance status; No., number; PD‐L1, programmed death‐ligand 1; SCC, squamous cell carcinoma; SRT, stereotactic radiotherapy.

### 3.3 Intracranial Therapeutic Response

The intracranial objective response rate (iORR) was significantly higher in the concurrent group compared to the sequential group (77.9% vs. 61.8%, respectively; *p* = 0.012). Similarly, the intracranial disease control rate (iDCR) was significantly higher in the concurrent group (91.2% vs. 79.4%; *p* = 0.026). Notably, among patients requiring corticosteroids or mannitol for symptom management, concurrent treatment was associated with a significantly better prognosis (*p* = 0.042) (Table 6).

**TABLE 6:** Outcomes in the matched datasets.

Projects	Median iPFS (months)	Median OS (months)	iORR (%)	iDCR (%)	1‐year iPFS (%)	1‐year OS (%)
Concurrent	14.2	24.5	77.9	91.2	67.6	85.3
Sequential	9.8	18.3	61.8	79.4	45.6	72.1
*p*‐value	< 0.001	0.001	0.012	0.026	0.003	0.021

Abbreviations: Concurrent, concurrent SRT + ICIs; iDCR, intracranial disease control rate; iORR, intracranial objective response rate; iPFS, intracranial progression‐free survival; OS, overall survival; Sequential, sequential SRT + ICIs.

### 3.4 Impact of Treatment Timing

Cox regression analysis treating treatment interval as a continuous variable revealed that each one‐week extension in the interval significantly increased the risk of intracranial progression by 18% (HR = 1.18, 95% CI: 1.05–1.33; *p* = 0.005) and the risk of death by 15% (HR = 1.15, 95% CI: 1.02–1.30; *p* = 0.024). The finding that a treatment interval ≤ 7 days was optimal aligns with results from a prior multi‐center retrospective study.

### 3.5 Treatment‐Related Toxicities

The combination of SRT and ICIs was well‐tolerated overall, with no new safety signals identified. The incidence of RN was low and comparable between the concurrent and sequential groups (7.4% versus. 5.9%, respectively; *p* = 0.832). The incidence of any‐grade immune‐related adverse events (irAEs) was also similar between groups (26.5% concurrent vs. 23.5% sequential; *p* = 0.648), as was the incidence of grade ≥ 3 irAEs (5.9% vs. 4.4%; *p* = 0.792). Treatment‐related pneumonitis (TRP) occurred in 11.8% of concurrent group patients and 10.3% of sequential group patients (*p* = 0.812). Multivariate analysis confirmed that treatment interval was not an independent predictor of TRP risk (*p* = 0.436).

## 4 Discussion

In this multi‐center retrospective analysis, we demonstrated the superior clinical efficacy of concurrent versus sequential SRT and ICIs for NSCLC patients with multiple BM. The results revealed that concurrent treatment significantly prolonged iPFS and OS, improved iORR, and did not increase treatment‐related toxicity risks. This finding provides valuable evidence for optimizing treatment strategies in NSCLC patients with multiple BM.

### 4.1 Synergistic Mechanisms and Timing Considerations of Combined Therapy

The combination of SRT and ICIs is based on a strong theoretical rationale. On one hand, radiotherapy can induce immunogenic cell death, releasing tumor‐associated antigens and damage‐associated molecular patterns (DAMPs). This process promotes antigen presentation and T‐cell activation and modulates the tumor microenvironment, thereby potentially enhancing the response to ICIs. On the other hand, radiotherapy may increase the permeability of the blood–brain barrier (BBB), facilitating improved central nervous system (CNS) penetration of ICIs [7, 8, 9, 10]. Studies indicate that ICIs alone exhibit moderate activity against brain metastases, with an iORR of approximately 20%–40% [4]. However, when combined with SRT, the iORR can be significantly enhanced, reaching up to 75.9% [11].

A key finding of this study is the significant impact of treatment timing on patient outcomes. Patients who received SRT and ICIs within a ≤ 14 days interval, especially ≤ 7 days interval, demonstrated significantly longer iPFS and OS compared to those with an interval > 14 days. This observation aligns with previous research, suggesting that concurrent or closely sequenced therapy may yield superior synergistic effects [12]. A potential mechanism is the existence of a critical time window of immune activation following radiotherapy, during which the administration of immunotherapy can maximally harness this synergistic anti‐tumor activity.

### 4.2 Impact of Treatment Sequence on Efficacy

The results of this study demonstrated that the concurrent therapy group achieved significantly superior median iPFS (14.2 months) and median OS (24.5 months) compared to the sequential group (9.8 months and 18.3 months, respectively). These findings are consistent with research presented at ESTRO 2024, which also reported longer OS for upfront SRS/SRT compared to upfront ICIs (median OS not reached versus. 14.6 months, *p* = 0.021) [6]. Another study similarly showed a marked OS advantage for upfront SRS/SRT versus upfront ICIs, with mean OS of 37.8 months versus 19.7 months (*p* = 0.021) [5]. Subgroup analysis revealed that the post‐SRT group achieved the best survival outcomes, with a 55%–67% lower mortality risk compared with the other three groups (all *p* < 0.01). Consistent with several prior retrospective cohorts investigating sequencing of SRT and ICIs for NSCLC brain metastases, our study demonstrated superior OS and iPFS in patients receiving ICIs after SRT (the post‐SRT of the concurrent group). The more prominent intergroup separation for iPFS relative to OS aligns with previous reports, possibly because overall survival is frequently confounded by extracranial progression competing risk events [5, 13, 14].

Notably, an analysis of the treatment interval as a continuous variable revealed that each one‐week extension of the interval was associated with an 18% increased risk of intracranial progression and a 15% increased risk of death. This indicated that treatment timing is a critical, continuous factor influencing the efficacy of the combination therapy, rather than a simple binary variable. In clinical practice, efforts should be made to minimize the interval between SRT and ICI administration to optimize treatment outcomes.

### 4.3 Safety and Tolerability

This study confirmed that the combination of SRT and ICIs has a manageable safety profile, with concurrent treatment not significantly increasing the risk of severe toxicities such as radiation necrosis. This is consistent with findings from multiple previous studies [15]. Several reports indicated that concurrent ICIs and SRS did not increase the risk of radiation necrosis [16, 17, 18]. Similarly, two comprehensive reviews suggested that the combination therapy was associated with significant overall survival benefits without a corresponding increase in radiation necrosis risk [19, 20].

Regarding the risk of radiation pneumonitis, this study found no significant influence of treatment timing on the incidence. This finding is not entirely consistent with some other studies, a discrepancy potentially attributable to differences in patient populations, radiotherapy techniques, and dose parameters [21, 22, 23, 24]. Overall, concurrent therapy maintains a manageable safety profile, not significantly increasing the incidence of severe toxicities while ensuring efficacy.

### 4.4 Study Limitations and Future Perspectives

This study has several limitations: (1) Its retrospective nature may introduce selection bias, despite the use of propensity score matching for adjustment; (2) Variations in treatment protocols and practices across participating centers might have influenced the outcomes; (3) The sample size was relatively limited, particularly for certain patient subgroups; (4) The follow‐up duration may be insufficient for the assessment of long‐term toxicities; (5) Subgroup analysis based on the specific type of ICIs administered was not performed.

Despite these limitations, the findings hold significant clinical relevance. For NSCLC patients with BM, concurrent SRT and ICIs represent an effective and relatively safe treatment option. Based on our results, it is recommended that clinical practice aims to initiate ICIs shortly after SRT (within ≤ 7 days) to maximize therapeutic benefit. Future research directions should include the conduct of prospective randomized controlled trials to definitively establish the optimal role of concurrent therapy in specific patient populations. Investigations into predictive biomarkers, such as PD‐L1 expression, tumor mutational burden, and features of the immune micro‐environment, are warranted. Furthermore, optimizing radiotherapy techniques and dose fractionation schedules to maximize synergy and minimize toxicity remains a crucial area of exploration.

## 5 Conclusion

This study demonstrates that concurrent SRT and ICIs (initiate ICIs shortly after SRT within ≤ 7 days) for NSCLC patients with multiple BM significantly improves iPFS, OS, and iORR compared to sequential therapy, without increasing the risk of treatment‐related toxicities. The treatment interval is a crucial prognostic factor, with shorter intervals (≤ 7 days) correlating with superior survival outcomes. These findings provide valuable evidence for personalizing treatment strategies for advanced NSCLC patients with BM. Prioritizing a concurrent treatment approach in clinical practice is recommended, as it holds promise for further improving the prognosis of this patient population.

## Author Contributions

Xiaoliang. Wang, Yanan. Zheng and Xiaolan. Zhao. wrote the main manuscript text and Aimin. Wang Qiong. Huang Kai. Guan Jiali. Du prepared figures and tables. All authors reviewed the manuscript.

## Funding

The authors have nothing to report.

## Ethics Statement

This study protocol was reviewed and approved by the Clinical Research Ethics Committee of the Third Hospital of Zhangzhou, Xiamen Changgung Hospital, and Army 73rd Group Military Hospital (Ethics Approval No.: Zhangzhou Medical Ethics 2025–005). All clinical procedures implemented in this study strictly complied with the principles outlined in the Declaration of Helsinki and its subsequent revisions. Written informed consent was acquired from all enrolled patients prior to data collection.

## Consent

Written informed consent for publication was obtained from all participants.

## Conflicts of Interest

The authors declare no conflicts of interest.

## Data Availability

The data that support the findings of this study are available from the corresponding author upon reasonable request.
